# Microglia and Wnt Pathways: Prospects for Inflammation in Alzheimer’s Disease

**DOI:** 10.3389/fnagi.2020.00110

**Published:** 2020-05-14

**Authors:** Yunying Yang, Zhentao Zhang

**Affiliations:** Department of Neurology, Renmin Hospital of Wuhan University, Wuhan, China

**Keywords:** Alzheimer’s disease, microglia, neuroinflammation, Wnt pathway, AD pathology

## Abstract

Alzheimer’s disease (AD) has been a major health issue for more than one century since it was first reported in 1906. As one of the most common neurodegenerative diseases, AD is characterized by the presence of senile plaques and neurofibrillary tangles (NFTs) in the affected brain area. Microglia are the major regulators of neuroinflammation in the brain, and neuroinflammation has become recognized as the core pathophysiological process of various neurodegenerative diseases. In the central nervous system (CNS), microglia play a dual role in AD development. For one thing, they degrade amyloid β (Aβ) to resist its deposition; for another, microglia release pro-inflammatory and inflammatory factors, contributing to neuroinflammation as well as the spreading of Aβ and tau pathology. Wnt pathways are important regulators of cell fate and cell activities. The dysregulation of Wnt pathways is responsible for both abnormal tau phosphorylation and synaptic loss in AD. Recent studies have also confirmed the regulatory effect of Wnt signaling on microglial inflammation. Thus, the study of microglia, Wnt pathways, and their possible interactions may open up a new direction for understanding the mechanisms of neuroinflammation in AD. In this review, we summarize the functions of microglia and Wnt pathways and their roles in AD in order to provide new ideas for understanding the pathogenesis of AD.

## Introduction

### Microglial Activation and Immune-Inflammatory Response in AD Development

**FIGURE 1:**
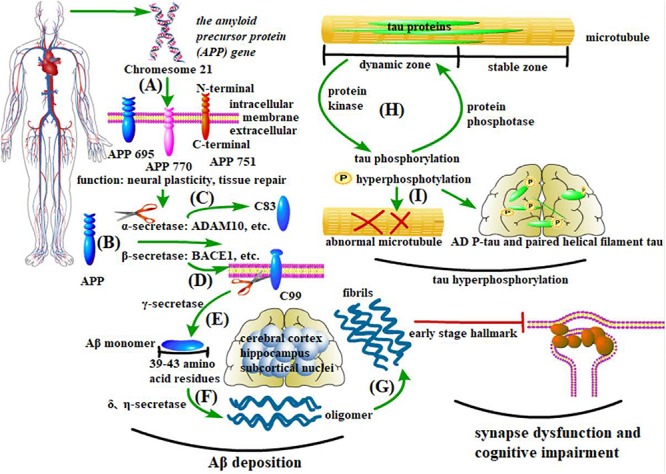
Pathological changes in AD. **(A–G)** Aβ deposition and synapse dysfunction: the *amyloid precursor protein* (*APP*) gene generates at least three isomers of APP proteins in the brain. β-secretase cleaves APP into the C-terminal fragment C99, which is sequentially processed by γ-secretase to produce Aβ. In contrast, α-secretase cleaves APP into C83, which inhibits Aβ deposition. Under pathological conditions, the soluble Aβ monomer turns into oligomers and insoluble fibers, with the former being more pathogenic. Existing in the cerebral cortex, hippocampus, and some subcortical nuclei, these anomalously aggregated Aβs cause synapse dysfunction, thus leading to cognitive impairment. **(H,I)** Tau protein phosphorylation: microtubules have both dynamic and stable zones, and tau mostly exists in the former. As major microtubule-associated proteins in nerve cells, the phosphorylation and dephosphorylation of tau proteins are controlled by protein kinases and protein phosphatases (PP), respectively. Abnormality of these processes will cause an aberrant structure of microtubules. In AD brains, two aberrant forms of tau proteins (AD P-tau and paired helical filament tau) are the main components of NFTs.

Amyloid-beta (Aβ) deposition, abnormal tau phosphorylation, and synaptic dysfunction are the characteristic pathological changes of AD (Figure 1; Robakis et al., 1987; Baas and Black, 1990; Wang et al., 1996; Klyubin et al., 2005; Shankar et al., 2008; Kuhn et al., 2010; Martin et al., 2013a, b; Pester et al., 2013; Zhang et al., 2014, 2018; Willem et al., 2015; Roostaei et al., 2017; Kreutzer and Nowick, 2018; Trillaud-Doppia and Boehm, 2018; Baas and Qiang, 2019). Tightly connected with AD pathology, microglia and microglial inflammation work actively in AD onset and progression. Secretion and phagocytosis are the most studied functions in microglial neuroinflammation. This is not only because microglia are the dominant immune cells releasing inflammatory or pro-inflammatory factors in the central nervous system; they also interact with other brain cells by secreting cytokines (Yang et al., 2011; Fricker et al., 2018). More importantly, various immunological receptors expressed on microglia have equipped them with the capability to proliferate, accumulate, change morphology, and, especially, phagocytose Aβ, so they function as both neuron protectors and injurers with activated neuroinflammation in AD development (Füger et al., 2017; Ayata et al., 2018). For example, CD22, an immunological receptor on microglia, has been newly proved to affect microglial phagocytosis to the greatest extent in aging brains (Pluvinage et al., 2019). As well as phagocytosis-related receptors, TREM2 is another important receptor on microglia that maintains cell function in AD and has been extensively studied lately from both the gene and protein level (Krasemann et al., 2017; Shi et al., 2017; Filipello et al., 2018; Sayed et al., 2018; Long et al., 2019; Yin et al., 2019; Zhou et al., 2019). TREM2 can inhibit β-catenin degradation while stabilizomg β-catenin, thus activating the canonical Wnt pathway to act protectively in AD. *TREM2* mutations will lead to abnormality of Wnt/β-catenin signaling and microglial dysfunction, which causes high risk of AD (Zheng et al., 2017; Meilandt et al., 2020). Thus, Wnt pathways and microglial functions may be the targets of some newly found genes that contribute to AD. The Wnt/β-catenin pathway will be described in detail in section Wnt Pathway Regulation Is Promising in AD Development.

### Wnt Pathway Regulation Is Promising in AD Development

Wnt signaling forms a vast network in the development, regeneration, and protumor pathogenesis of all the vital organs in human body (Watson et al., 2013; Kim et al., 2017; Patel et al., 2017; Shen et al., 2017; Ma et al., 2019). In the CNS, Wnt signals contribute to vascular repair and the development of the blood-brain barrier and share communications with microglial neuroinflammation (Hübner et al., 2018; Van Steenwinckel et al., 2019). Wnt signals are composed of one canonical pathway (Wnt/β-catenin) and two non-canonical pathways (Wnt/PCP and Wnt/Ca^2+^), as well as their cascade molecules. Targeting AD pathology, all these pathways are indicated in AD development (Elliott et al., 2018; Amal et al., 2019; Petrache et al., 2019).

The canonical Wnt pathway centers on the core molecule – β-catenin. β-catenin can be normally degraded through ubiquitination, while Wnt3a, a member of the canonical pathway, can stabilize β-catenin. When Wnt proteins of canonical families combine with their receptors FZD and LRP5/6 on microglia, β-catenin then accumulates in the plasma, transfers to the nucleus, and binds to the TCF/LEF transcription factor, thus initiating the transcription of the targeted genes (Halleskog et al., 2011; Gray et al., 2013; Ren et al., 2015). This activation of the Wnt canonical pathway changes the immune-phenotype of microglia and brings microglia into an activated and pro-inflammatory state, which is symbolized by high expression of IBA1, CD68, CD33, and CD11b (i.e., CR3) (Halleskog and Schulte, 2013; Mrdjen et al., 2018; Böttcher et al., 2019; O’Koren et al., 2019). Microglial activation then alleviates AD pathology, and this again proves the dual role microglial neuroinflammation plays in AD development, since neuroinflammation is not completely negative for protecting normal brains from AD (Toledo and Inestrosa, 2010). However, according to the literature available at present, whether Wnt signaling has a direct influence on microglial phagocytosis remains unknown; microglial pro-inflammatory transmission is currently thought to be the main target that Wnt signaling relies on to exert effect (Song et al., 2019; Van Steenwinckel et al., 2019). There is no doubt, however, that microglial phagocytosis is always followed by the presence of inflammation. Therefore, investigating not only the Wnt-related microglial inflammation but also the effect of Wnt signaling on the other microglial functions such as phagocytosis is of great significance. Although there is no evidence supporting the direct interaction between Aβ and Wnt proteins, Aβ is believed to promote β-catenin phosphorylation and cause β-catenin degradation and to still be able to block Wnt signaling in an indirect way by combining with the cysteine-rich region of Wnt receptor FZD (Chen and Bodles, 2007; Magdesian et al., 2008; He and Shen, 2009). In AD models, a sharp decrease of β-catenin and Aβ overproduction inhibit the canonical Wnt pathway and further decrease Wnt receptors (Liu et al., 2014; Elliott et al., 2018).

**FIGURE 2:**
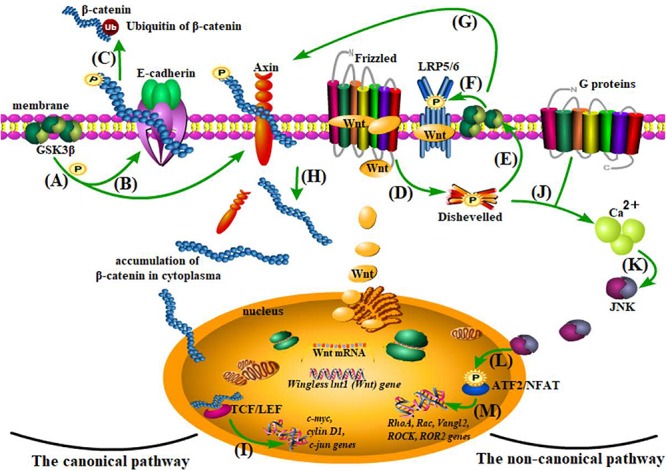
The Wnt pathways. **(A–C)** β-catenin degradation: under normal conditions, β-catenin is anchored to the cell membrane with E-cadherin and Axin and goes to ubiquitination after being phosphorylated by glycogen synthase kinase 3 (Gsk3β). **(D–I)** The canonical pathway: when Wnt combines with the cysteine-rich region of its receptor protein, Frizzled (FZD), and the coreceptor-low density lipoprotein receptor-related protein 5/6 (LRP5/6) acts on the Disheveled (Dvl) protein and inhibits Gsk3β, thus separating β-catenin from Axin, the accumulated β-catenin from plasma to nucleus is capable of enhancing the activity of TCF/LEF (a transcriptional factor) and therefore influencing gene transcription of *c-myc*, *cylin D1*, *c-jun*, etc. **(J–M)** The non-canonical pathway: the activation of the Wnt/planar cell polarity (PCP) and the Wnt/Ca^2+^ pathways, although independent of β-catenin, share the initial process (Wnt combines with receptors and activates Dvl) with the canonical pathway. With the assistance of G proteins, Ca^2+^ is recruited in the plasma to cause cascade reactions and finally affect transcription of *Rac*, *RhoA*, *Vangl2*, *ROCK*, and *ROR2*.

The two non-canonical Wnt pathways (members: Wnt11, Wnt5a, Wnt7b, Wnt9b, etc.) are independent of β-catenin (Figure 2) and are best known for regulating cell fate (differentiation, proliferation, etc.) and cell activities (polarization, migration, etc.) (Rosso et al., 2005; Ciruna et al., 2006; Carmona-Fontaine et al., 2008; Farías et al., 2009; Ishida-Takagishi et al., 2012; Florian et al., 2013; Scholz et al., 2016; Chavali et al., 2018). Wnt5a, one of the members of the non-canonical Wnt pathways, is also proved to contribute to microglial inflammation and AD development (Farías et al., 2009; Kitazawa et al., 2011; Killick et al., 2014; Sellers et al., 2018), the underlying mechanism of which has not been discovered.

**FIGURE 3:**
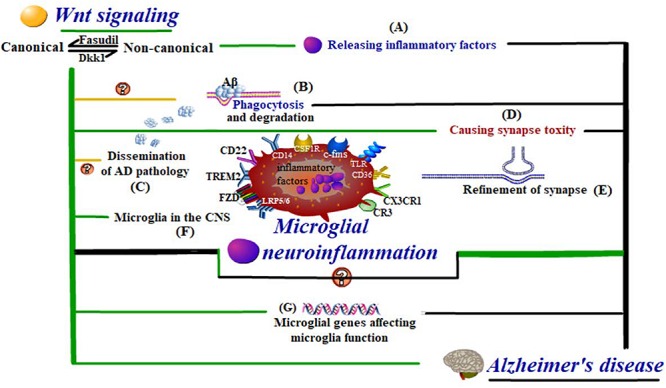
Wnt-regulated microglial neuroinflammation in Alzheimer’s disease. Wnt regulation influences the cell fate and activities of **(F)** microglia in the CNS, even at the level of **(G)** microglial genes affecting microglial functions. These functions include **(A)** inflammatory response, **(B)** Aβ phagocytosis and degradation, **(C)** dissemination of AD pathology, and **(D,E)** synapse modification, which are all closely related to neuroinflammation in AD development.

In summary, the activation of canonical and non-canonical pathways can both lead to microglial inflammation, with the former playing a protective role in AD development and the latter contributing to AD development. More often than not, whilst the canonical pathway is restrained, the non-canonical one will be activated. The inhibitor of the canonical pathway and activator of non-canonical pathways, Dickkopf-1 (Dkk1), which is produced by Aβ induction, is proved to cause synaptic toxicity (Killick et al., 2014). In contrast, Fasudil, the inhibitor of the non-canonical Wnt pathways, is proved to effectively reverse the unbalance between canonical and non-canonical Wnt pathways and reduce the synaptic toxicity caused by Aβ (Sellers et al., 2018). However, the mechanism by which the shift from canonical to non-canonical Wnt pathway transmits to alter the role of microglia from immune executors to neuron injurers is still under investigation (Figure 3). It can be speculated that Wnt signaling, no matter whether from inside microglia or from other brain cells, must affect the cell fate, activities, or functions of microglia to influence microglial neuroinflammation and thus be involved in AD, possibly at the transcriptional, gene, or molecule level. Controversially, some research has adopted interventions to attenuate microglial inflammation via activating the canonical Wnt pathway, which has added complexity to the determination of the relationship between the canonical Wnt pathway and microglial inflammation (Chen et al., 2017; Jiang et al., 2017; Bhat et al., 2018; Yao et al., 2019; Zhang D. et al., 2019).

## Microglia are More than Immune Cells in the Central Nervous System

The existing literature has confirmed the regulatory effect of Wnt pathways on microglial inflammation, and Wnt-targeted interventions such as Fasudil are promising in AD treatment. However, these results are far from explaining in detail the intermediate elements prior to the ultimate pro-inflammatory effect, which gives rise to limitations to the development of Wnt-targeted or microglia-pertinent therapies for treating AD. These intermediate elements cover three aspects: cell fate (microglial development in the CNS), cell activities (polarization, migration, and correlations with other cells) and cell functions (phagocytosis, release of inflammatory factors, refinement of synapses, and microglial genes affecting functions) of microglia. Therefore, attention has been focused on explaining how Wnt signaling affects the three aspects or follows other mechanisms, thus regulating microglial neuroinflammation in AD development. In sections Microglia Are More Than Immune Cells in the Central Nervous System, Microglial Aβ Phagocytosis-Degradation-Dissemination and Concomitant Inflammation, and Wnt promotes Microglial Inflammation in AD Development, we will discuss the important aspects around microglia in the hope of finding the potential targets of Wnt signaling on microglia.

### Microglia Participate in CNS Development, Conduct Synapse Modification, and Crosstalk With Wnt Signaling

The development of the CNS can hardly occur without microglia. During brain development, microglia will release growth-promoting substances to assist the neurogenesis of the hippocampus and cortex and promote the maturity of neural progenitor cells (NPCs) via certain physiological processes (Choi et al., 2008; Sierra et al., 2010; Ueno et al., 2013). Conversely, NPCs can guide the migration and accumulation of microglia in the subventricular zone (SVZ) (Arnò et al., 2014). However, the relationship between microglia and neurons is much more complex than that: the effect microglia have on synapses and neural circuits is multilevel. From the perspective of neurogenesis, microglia can act as synapse pruners by way of phagocytosis based on some immunological receptor pathways, the representative one of which is the CR3/CX3CL1 molecule pair on microglia (Barger and Basile, 2001; Paolicelli et al., 2017; Gunner et al., 2019). Under the intervention of harmful stimulus, the chemokine CX3CL1 is transformed by ADAM10 into its secretory form, interacts with its receptor Fractalkine specifically expressed on the neurons, and thus protects synapse function. This physiological refinement is conducive to maintaining the normal number of synapses and promoting the maturation of neuronal circuits (Schafer et al., 2012). When the canonical Wnt pathway is inhibited, Fractalkine expression will undergo a sharp disease, thus causing synapse degeneration (Gunner et al., 2019; Ru et al., 2019). This corresponds with the fact that Wnt3a (a member of the canonical Wnt pathway) upregulation and Wnt5a (a member of the non-canonical Wnt pathways) downregulation promote neurogenesis (Joseph et al., 2017; Bhat et al., 2018). Therefore, Wnt signaling may play a role in microglial synapse modification, and whether this sequentially contributes to synchronous microglial inflammation is worth investigating.

From the other perspective, when the CNS is confronted with neurodegenerative events such as during AD development, the abnormal expression of tau protein can enhance the combination of the complement fragment C1q and synapses and, as a result, over-activate microglial phagocytosis of synapses (Dejanovic et al., 2018; Bie et al., 2019). Aβ is sure to cause synaptic toxicity and then be phagocytosed by microglia when Aβ itself accumulates to a higher concentration, the mechanism of which will be discussed concretely in section Microglial Aβ Phagocytosis-Degradation-Dissemination and Concomitant Inflammation. Once the synapses are damaged by Aβ, they will be eliminated by microglia. Meanwhile, during this process, deleterious substances produced from microglia can simultaneously induce synapse dysfunction, which is the pathological foundation of cognitive impairment in AD and, to some extent, decides the AD plaque burden a patient can bear (Robinson et al., 2014; Turano et al., 2015; Edwards, 2019). These deleterious substances produced from microglia are from at least two sources: one is the harmful compounds released during microglial synapse elimination, while the other is the inflammatory factors delivered in step with the process of microglial Aβ phagocytosis (Jiang et al., 2016). As a matter of fact, Aβ not only damages synapses directly in this way but it also combines with the Wnt receptor FZD, thus causing the dysfunction of the canonical pathway (Magdesian et al., 2008). What makes this inhibition effect worse is that Aβ can also induce the production of the powerful inhibitor of the canonical Wnt pathway, Dkk1, which further inactivates this protective pathway against AD (Killick et al., 2014). This no doubt will aggravate the microglial inflammation and synapse damage, which is detrimental for reversal of AD, because the microglial synapse modification is the initiator rather than the consequence of AD and happens as early as in the initial stage of AD, just before NFT formation (Yoshiyama et al., 2007; Lui et al., 2016; Sellgren et al., 2019).

### Regeneration vs. Degeneration: Wnt-Regulated Microglial Activities in AD

So far, we have shown that despite microglia not being constitutive components of the CNS, they are still closely tied with NPCs, neurons, and other glial cells (Xiong et al., 2016). As immunological executors in the CNS, microglia are more than immune cells (Morizane et al., 2017). Since the merit of Wnt pathways in tissue regeneration has been widely noticed, microglia also play a role in neural regeneration through their correlations with neurons, oligodendrocytes, and astrocytes (Dou et al., 2012; Li et al., 2012; Slotkin et al., 2017; Wlodarczyk et al., 2017; Giera et al., 2018; Hill et al., 2018; Bellver-Landete et al., 2019). Even in an ALS model, astrocytes are considered to promote microglial proliferation along with neural protective effects in a Wnt-dependent way (Ouali Alami et al., 2018). Although the canonical Wnt pathway and proper content of microglial activation act protectively in neurogenesis, these two protective effects are not always in step with each other (Bhat et al., 2018). Some extreme situations can better prove these correlations. The Wnt3a-stimulated glioblastoma cells, which can be seen as the result of overaction of the canonical Wnt pathway, show greater invasiveness than general glioblastoma and greater microglial infiltration when co-cultured with microglia (Matias et al., 2019). In this case, Wnt3a is again proved to be able to change the immune phenotype of microglia, which is likely to produce inflammation rather than promote neural repair (Matias et al., 2019; Song et al., 2019; Van Steenwinckel et al., 2019). These results have demonstrated that Wnt signaling is a double-edged sword to microglial fate and activities, which is similar to the fact that microglial neuroinflammation is also a double-edged sword in neurodegenerative diseases, including AD. In the cases of cell aging and neurodegenerative diseases, microglia will change into an activated state, resisting pathological alternation of these diseases while simultaneously accelerating neurodegeneration via immune and inflammatory response (Davies et al., 2017; Gibson et al., 2019). These immune responses show different levels of severity in different brain regions, with the cerebellum and hippocampus being most sensitive, which may account for the phenomenon that some pathological proteins specifically concentrate on certain brain regions (Grabert et al., 2016). This bidirectional effect of Wnt pathways on microglia and the multilevel effect of microglial inflammation on AD development require us to adopt appropriate interventions to regulate Wnt pathways, in expectation of controlling the microglial activation and inflammation at the level of neural repair and protection rather than neurodegeneration.

### Exploring Elements Affecting Microglial Functions at the Gene Level and Gene-Level Wnt Regulation

At the end of section Regeneration vs. Degeneration: Wnt Regulated Microglial Activities in AD, we put forward a goal of Wnt regulation, which involves looking for a balance of Wnt-regulated microglial inflammation in AD. The complexity of this goal lies in that microglia share an extensive connection with neurons and other glial cells in the CNS, which means that microglial inflammation participates not only in AD but in other neurodegenerative diseases. Moreover, Wnt signaling itself has a wide range of effects inside the human body. However, the present studies concerning the relation between Wnt signaling and microglia have not covered the non-canonical Wnt pathways, and even the regulation of the canonical pathway on microglia is still stagnating at the level of biological effect (pro-inflammatory effect). To make this goal feasible, strict regulation at the molecular level, gene level, and even transcriptional level is greatly needed. No matter what the microglial biological effects are, modifying synapses or promoting inflammation, these effects are closely related to phagocytosis and other microglial functions, which are based on the corresponding immunological receptors or other molecules. These biological factors also affect the microglial function at the gene level, the effect of which will be greatly disturbed when certain gene mutations happen in microglia (Schafer et al., 2016; Nagarajan et al., 2018). TREM2 is such an important receptor on microglia, maintaining cell function and affecting Aβ processing in AD (Ulland et al., 2017). This receptor is capable of stabilizing β-catenin, thus activating the canonical Wnt pathway to act protectively in AD. *TREM2* mutation or deletion will lead to abnormality of Wnt/β-catenin signaling and microglial dysfunction, which causes a high risk of AD (Zheng et al., 2017; Meilandt et al., 2020). What is more, the mutation of the classical AD-susceptible gene *PSEN* was proved early on to be followed with β-catenin upregulation (Zhang et al., 1998). Aside from this, with the increase in focus on the biological effects of competing endogenous RNA (ceRNA, a collection of non-coding RNAs over 200nt interacting with mRNA, thus influencing gene expression, with miRNA, rRNA, lncRNA, circRNA, etc. included), recent studies have explored the Wnt regulation on microglia affected by some lncRNA, which opens up a new direction for studying Wnt regulation on microglia at the gene level (Xia et al., 2017; Ross et al., 2018; Cherubini et al., 2019; Han and Zhou, 2019; Zhang L. et al., 2019). Considering that many neurodegenerative diseases possess genetic susceptibility where genes concerning microglial functions are involved, the pathological changes of various neurodegenerative diseases related to these genes and corresponding microglial functions are presented in Table 1. These may provide possible targets for Wnt regulation on microglia at the gene level (Chrétien et al., 2004; Wiendl et al., 2005; Baker et al., 2006; Bensinger and Tontonoz, 2009; Llorens et al., 2014; Karch and Goate, 2015; Markovinovic et al., 2017; Aliseychik et al., 2018; Conway et al., 2018; Rui et al., 2018; Crotti et al., 2019; Estus et al., 2019; Filippini et al., 2019; Henstridge et al., 2019; Huang et al., 2019; Sakae et al., 2019).

**TABLE 1:** Microglial genes contributing to neurodegenerative diseases.

**Diseases**	**Microglial genes**	**Mechanism pathology/function**	**Main clinical symptoms**
Alzheimer’s disease (AD)	TREM1/2	Immune response, Aβ phagocytosis, synapse modification and related to tau pathology	Cognitive impairment
	APOE4	Lipid metabolism, Aβ deposition	
	PLCG/ABI3	Immune response, Aβ pathology	
	PSEN	Immune response, Wnt pathway	
	PTK2B/PICALM/CR1	Endocytosis, Aβ metabolism	
	CD22/14/36/33	Immune response, Aβ phagocytosis	
	HLA	Immune response	
	BIN1	Endocytosis, tau pathology	
	CR3	Immune response, synapse elimination	
	HDAC1/2	Aβ phagocytosis	
Parkinson’s disease (PD)	Nurr1	protection of dopaminergic neurons	Resting tremor
	MHC II	Immune response	
	α-syn	Spreading of misfolded proteins	
	LRRK2	Accumulation of α-synuclein	
Amyotrophic lateral sclerosis (ALS)	TBK1/p62	Axonal degeneration	Immobility
Multiple system atrophy (MSA)	PLCG/ABI3	Immune response	Similar to early PD
Creutzfeldt-Jakob disease (CJD)	Pro-inflammatory cytokines, etc.	Neuroinflammatory response	Compound performances
	PGRN	Neuronal survival	
	EAAT-1	Neuroprotective function	

## Microglial Aβ Phagocytosis-Degradation-Dissemination and Concomitant Inflammation

**FIGURE 4:**
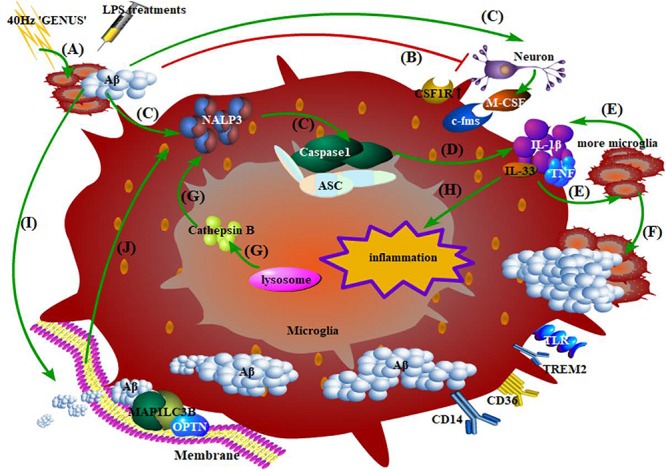
Phagocytosis, degradation of Aβ by microglia, and the inflammatory response. **(A)** 40 Hz “GENUS” induces Aβ accumulation, while LPS treatments regulate immunologic memory; **(B)** Aβ at high concentration directly damages the neurons; **(C)** early events of microglia-mediated inflammation: Aβ-induced combination of endogenous substances in neurons and their upregulated receptors on microglia, accumulation of NALP3 inflammasome, and activation of caspase-1 precursor; **(D)** microglia release inflammatory factors; **(E)** inflammatory factors recruit more microglia with more production of neurotoxic factors; **(F)** microglia migrate to, surround, and phagocytose Aβ; **(G)** cathepsin B released from damaged lysosomes in microglia directly reinforces NALP3 accumulation; **(H)** inflammatory factors produced during this period promote neuroinflammation; **(I)** Aβ activates the initiation of autophagy and membrane extension; Aβ compounds are formed and then degraded via the STK11/PRKAA1 pathway; **(J)** An outbreak of autophagy, in turn, promotes the accumulation of the abovementioned caspase-1 and certain inflammasomes.

Except for regulating synaptic functions, microglia are also capable of regulating Aβ deposition by phagocytosis. Since microglia share an extensive connection with neurons based on some special extracellular micro-vesicles, the proper processing of Aβ by microglial phagocytosis seems to be of great significance in that microglial dysfunction will also be harmful for neurons (Budnik et al., 2016; Vogel et al., 2018; Wheeler et al., 2018; Klein et al., 2019). A high concentration of Aβ has a direct toxic influence on neurons, while a low concentration induces the exposure of signals on neurons and attracts microglia to phagocytose and degrade Aβ (Neniskyte et al., 2011; Wendeln et al., 2018). Under Aβ induction, neurons release endogenous substances to combine with their upregulated receptors on microglia, which is followed by pro-inflammatory events such as inflammasome accumulation in microglia (Du Yan et al., 1997; Olmos-Alonso et al., 2016). This is followed by the release of inflammatory factors like IL-1β, IL-33, and COX2, which recruits much more microglia with increasing production of other neurotoxic factors (Halle et al., 2008; Johansson et al., 2015; Fu et al., 2016; Lee J.Y. et al., 2018). Under chemotaxis, these microglia migrate to and surround their targets – Aβ and tau – and engulf them through special pathways and receptors such as CD14 and CD36 (Liu et al., 2005; Lucin et al., 2013; Chen et al., 2016; Jiang et al., 2016; Martin et al., 2017; Stancu et al., 2019). The inflammatory factors produced during this period will further reinforce inflammasome accumulation, forming a vicious circle (Halle et al., 2008). Sequentially, Aβ is degraded after the membrane extension and autophagy of microglia are initiated, which in turn will promote the abovementioned pro-inflammatory events (Figure 4; Cho et al., 2014; Halle et al., 2008). The microglial phagocytosis is negatively regulated by CD22 but positively regulated by TREM2, 40 Hz gamma entrainment using sensory stimulus (“GENUS”), and *HDAC1/2* knockout (Datta et al., 2018; Martorell et al., 2019; Parhizkar et al., 2019; Pluvinage et al., 2019).

The already known effect of Aβ on Wnt pathways has two aspects. One is that Aβ and APP promote β-catenin phosphorylation and degradation, thus inhibiting the canonical Wnt pathway (Kim et al., 2003; Chen and Bodles, 2007; He and Shen, 2009). Tau protein is believed to stabilize β-catenin so that it can resist degradation, and the abnormal modification of tau can also cause damage to the canonical Wnt pathway (Liu et al., 2020). Another aspect is that Aβ can combine with the extracellular cysteine-rich domain of Wnt receptor FZD (a receptor in the canonical pathway), and APP shares co-binding domains with Wnt co-receptor LRP6 (a receptor in the non-canonical pathways) (Magdesian et al., 2008). When LRP6 binds to the E2 domain of APP, APP will be less cleaved by secretases, thus attenuating Aβ deposition (Lin et al., 2011; Liu et al., 2014; Elliott et al., 2018). Therefore, Aβ has an influence not only on the canonical Wnt pathway but also on the non-canonical ones. Wnt signaling dysfunction causes Aβ production and synapse loss and adds difficulty to neurogenesis (He and Shen, 2009; Elliott et al., 2018). Simultaneously, Aβ will induce Dkk1 production, which further inhibits the canonical Wnt pathway to exacerbate AD pathology (Killick et al., 2014). The increasing Aβ burden, together with Wnt signaling dysfunction, will place great pressure to microglia. On the one hand, microglia will release various inflammatory factors when phagocytosing Aβ; on the other hand, Wnt signaling dysfunction will impair microglial synapse modification and promote microglial inflammation (Ru et al., 2019; Song et al., 2019; Van Steenwinckel et al., 2019).

Whether the microglial function of phagocytosis will be directly injured by abnormal Wnt regulation under the double enhancement of inflammation needs to be further explored. In any case, phagocytosis is a pivotal aspect for microglia, affecting microglial neural repair, synapse modification, and inflammation profoundly. Aβ and tau dissemination may be a manifestation of microglial phagocytic overload, as mentioned above. When Aβ phagocytosis exceeds degradation, Aβ is more likely to disseminate in the brain, thus enhancing inflammation. At present, ASC specks, which act as the receptor of inflammasome, are considered to be responsible for Aβ dissemination (Halle et al., 2008; Freeman et al., 2017; Venegas et al., 2017). This proves that inflammation is also responsible for the dissemination of AD pathology, and it follows that the balance of phagocytosis-degradation is rather important for microglial functions in AD; otherwise, microglial phagocytosis will be favorable for tau and Aβ dissemination (Asai et al., 2015). The biological basis of this phenomenon is still elusive. During this process, active Wnt proteins may act as the messenger, carried by the extracellular vesicle exosome. Exosome can be secreted by cells of all kinds, including microglia (Gross et al., 2012; Hooper et al., 2012; Ibrahim et al., 2019; Kaiser et al., 2019). Wn3a is able to induce exosome secretion from microglia (Hooper et al., 2012). Evidence has shown that depletion of microglia and inhibition of exosome synthesis halt tau propagation (Asai et al., 2015). Also, Wnt proteins secreted on exosomes by neurons are capable of regulating synapse numbers; however, the relationship between this regulation of synapses and microglial synapse modification is still under investigation (Luga et al., 2012; Lee S.H. et al., 2018). This indicates the possibility that not only Wnt pathways but also Wnt proteins themselves have an influence on microglial functions. Therefore, exploring the structural connection between Aβ (or tau) and Wnt proteins inside or outside microglia may further reveal the mechanism of Wnt-related microglial functions. Follow-up studies may concentrate on enhancing Aβ phagocytosis and degradation by microglia, balancing the two processes, preventing AD pathology dissemination, and antagonizing inflammation.

## Wnt Promotes Microglial Inflammation in AD Development

Wnt5a (a component of non-canonical Wnt pathways) and Wnt3a (a component of the canonical Wnt pathway) have been most studied for microglial Wnt regulation. As mentioned in the Introduction section, although the canonical Wnt pathway is believed to act protectively while the non-canonical ones act deleteriously in AD, they are both responsible for microglial inflammation. Some studies have shown that activation of the canonical Wnt pathway prevents microglial activation and alleviates neuroinflammation (Bhat et al., 2018; Song et al., 2019; Zhang D. et al., 2019). However, in primary microglia, activated Wnt-3a can upregulate β-catenin in the microglia and bring it into a pro-inflammatory state in AD (Kilander et al., 2011; Hooper et al., 2012; Halleskog and Schulte, 2013). A paradox therefore exists in that Wnt3a, as a member of the canonical Wnt pathway, which is supposed to prevent microglial inflammation in AD, conversely promotes it. This no doubt shows the complexity of Wnt regulation in microglial inflammation. However, a deeper understanding of Wnt-related microglial inflammation targeting microglial neurogenesis, synapse modification, and phagocytosis-degradation-dissemination of AD pathology has not been obtained as yet. It can be speculated that the canonical Wnt pathway is capable of maintaining microglial inflammation at a level conducive to resisting AD pathology, while both canonical Wnt pathway overactivation and the non-canonical Wnt pathways aggravate microglial inflammation, which further exacerbates AD pathology (Dijksterhuis et al., 2015). During this process, Wnt regulation of the specific microglial functions is worth exploring, especially the non-canonical pathways, which have not been covered completely.

## Discussion

At present, the mechanism of AD has not been fully revealed, and microglial functions will need to be taken into consideration to bring the AD animal models used in researches much closer to the real conditions. Wnt signaling is a promising and powerful tool for resisting AD development. Based on the devotion of both microglia and Wnt pathways to AD development, future directions for AD pathogenesis concerning microglial inflammatory response via the Wnt pathway have been put forward in our review in sections Microglia Are More Than Immune Cells in the Central Nervous System, Microglial Aβ Phagocytosis-Degradation-Dissemination and Concomitant Inflammation, and Wnt promotes Microglial Inflammation in AD Development. Given that the microglial immune-inflammatory response, Wnt-mediated cell development, and formation of AD pathology are all chronic and progressive processes, figuring out the interrelations among them is of significance to further reveal the pathogenesis of AD and even other neurodegenerative diseases. Although functions of Wnt pathways in cancer and embryonic development have already been discovered, the influence they have on microglia and AD remains to be explored. What is more, the production of inflammatory factors from microglia is susceptible to activation of the non-canonical Wnt pathway, the mechanism of which is worth investigating. Taken together, possible subjects for further studies are as follows: (1) effects of Wnt signaling on microglial cell fate, activities, and functions; (2) effects of non-canonical Wnt pathways on microglial neuroinflammation; (3) mechanism of the shift between the canonical and non-canonical Wnt pathways and how this affects AD development; (4) whether the shift between the canonical and non-canonical Wnt pathways is responsible for the dual effects of microglial neuroinflammation in neurodegenerative diseases; (5) the balance and mechanism of phagocytosis degradation of microglia and their interplay with inflammation in AD; (6) the biological basis and mechanism of Wnt-induced AD pathology dissemination by microglia; (7) the relation between Aβ and Wnt in the protein structure; (8) Wnt-pertinent drugs and microglia-pertinent therapies for AD treatments.

## Author Contributions

YY and ZZ provided the idea, reviewed the documents, and completed the manuscript.

## Conflict of Interest

The authors declare that the research was conducted in the absence of any commercial or financial relationships that could be construed as a potential conflict of interest.

## Abbreviations

AD: Alzheimer’s disease
NFTs: neurofibrillary tangles
CNS: central nervous system
A β: amyloid β
APP: amyloid precursor protein
PP: protein phosphatases
Dkk1: Dickkopf-1
Gsk3 β: glycogen synthase kinase 3
FZD: Frizzled
LRP5/6: low-density lipoprotein receptor-related protein 5/6
Dvl: Disheveled
PCP: planar cell polarity
NPCs: neural progenitor cells
SVZ: subventricular zone.
